# Prohibition or Freedom

**Published:** 1919-07-19

**Authors:** 


### PROHIBITION OR FREEDOM.

In the heat of controversy, while extremists are battling for prohibition on the one hand and for removal of all restrictions on the other, certain fundamental principles involved are liable to be forgotten, and the moderate man may be excused if he is torn now in one direction and now in another by exaggerated statements purporting to be facts, statements which those who are looking forward to genuine reform greatly regret.

The fundamental principles referred to are the law of natural selection, or survival of the fittest, and the truly democratic principle that all lasting reforms must come from within the individual. In the case of natural selection one is apt to think that it applies only to that part of our nature which we possess in common with the lower animals, and that it concerns itself solely with the question of physical superiority, or brute force, to the exclusion of mental qualities. But Darwin showed that even among the lower animals any attribute which tends towards the preservation of the species, such as cunning, is fostered and carefully preserved by Nature, and this indicates that all those higher attributes of the mind which belong to man, such as morality, truthfulness, and many other intellectual qualities that are beneficial in maintaining and improving the race, are utilised by Nature in her process of selection. It takes untold ages to form a species, and Professor Keith has shown that even man himself is not altogether of mushroom growth, but has existed on the earth nearly a million years, in physical appearance much as we know him to-day. And what has Nature been doing all this time? Certainly not standing still and looking on, but selecting the race carefully by a gradual accumulation of beneficial qualities. During all this long period man has been surrounded by enemies. In the early stages these were the elemental forces of Nature and the animal world, which he subdued by skill and cunning; and later, as civilisation advanced, the enemies came from the vegetable world in the shape of disease germs, which are still unsubdued; while in the last stage, the beginning of which probably extends much farther back than the historical period, alcohol was added, to subdue which we must proceed as we do against disease and raise the immunity of the individual—his power of resistance, or will power. The task of totally abolishing alcohol from the earth is like trying to abolish disease germs—a task which science has given up, and we are therefore thrown back on fortifying the individual, which is the only true reform.

Abuse of alcohol is universally condemned as harmful, and Nature recognises this in that alcoholics tend to die out, and anyone who surveys the historical period in England alone must recognise that we have become a soberer nation not by legislation, but by conviction. And this brings me to my second principle—that reform must proceed from within.

If it is a fact that alcohol, taken in moderation, is harmful, and will lead to race deterioration and eventual extinction, it is obvious that only those races will survive which adopt teetotalism; but that alcohol in all forms and in moderate quantity is harmful has yet to be proved, and unfortunately it is that part of the community in the best position to judge of the effect of alcohol on the human race—viz., the medical profession—which is unable to supply the definite information. In common with laymen, they recognise the terrible results of excess; but on the question of moderation they are hopelessly divided, a small minority believing that alcohol in any quantity is an evil in health or disease, and a large majority that, taken at proper times and quantities, it is valuable in health and indispensable in disease.

Looking at the question in its broad or racial aspect, it is unfortunate for the advocates of teetotalism that the dominant races just now are not the teetotal ones, whatever the future may bring forth. The harmfulness or otherwise of a moderate use of alcohol is a question that must be definitely settled before the question of prohibition can be finally answered, and it is our firm belief that, if it were definitely proved that the moderate use of alcohol is injurious to the health of mind and body, the common-sense of mankind would settle the question of abstention, for where there is no demand there is no supply. There is a Chinese proverb which says that a nation with many laws is in a bad condition, and there is great truth in this. We pride ourselves as a nation on our individual freedom, and that we do instinctively many actions for the common good which other nations will only do by regulation; it is our opinion that it is better to have a nation free to take alcohol, but sober from conviction, than a nation which can only be kept in bounds by prohibition and can never be trusted to remain sober when temptation occurs. In the first case you have a sturdy, independent race, and in the other a race of slaves, little better than sheep. Can you imagine such a race inheriting the earth? Why has the Anglo-Saxon race dominated the globe? Because wherever they go they have carried with them the great qualities of their race—viz., individuality and freedom and the capacity of independent action in emergencies. And so in this controversy do not let us lose our balance or sense of proportion, but go on fostering national reforms, as we have done in the past, by educating the individual to act and think for himself, for in this way only lies true reform.

